# Saliva culture as a predictive indicator for current blood infections and antimicrobial resistance in the ICU setting

**DOI:** 10.1038/s41598-023-47143-3

**Published:** 2023-11-20

**Authors:** Leonardo Moura Brasil da Rocha Santos, Lucas de Paula Ramos, Carlos Eduardo Rocha Santos, Diego Garcia Miranda, Mariana Gadelho Gimenez, Vanessa Marques Meccatti, Amjad Abu Hasna, Marcela dos Santos Oliveira, Morun Bernardino Neto, Luciane Dias de Oliveira

**Affiliations:** 1 Department of Biosciences and Oral Diagnosis, Institute of Science and Technology, São Paulo State University (ICT-UNESP), Francisco José Longo 777, São Dimas, São José dos Campos, SP 12245-000 Brazil; 2 Instituto Policlin de Ensino e Pesquisas-IPEP, Av. Nove de Julho, 430-Vila Ady’Anna, São José dos Campos, SP 12243-001 Brazil; 3 Laboratory “Systemic Health Care”, EA4129, University of Lyon, Lyon, France; 4 Present Address: UFR de Médicine, Université Lyon 1, Lyon, France; 5 Laboratoire des Multimatériaux et Interfaces CNRS (UMR 5615), Université Lyon 1, Villeurbanne, France; 6 Department of Restorative Dentistry, Endodontics Division, Institute of Science and Technology, São Paulo State University (ICT-UNESP), Av. Francisco José Longo 777, São Dimas, São José dos Campos, SP 12245-000 Brazil; 7 Anhembi Morumbi University, Benedito Matarazzo, 6070-Jardim Aquarius, São José dos Campos, SP 12230-002 Brazil; 8 Departamento de Ciências Básicas e Ambientais-LOB, Escola de Engenharia de Lorena-EEL/USP, Estrada Municipal do Campinho, s/no, Lorena, SP 12602-810 Brazil

**Keywords:** Diseases, Health care, Medical research

## Abstract

Antimicrobial resistance is a worldwide health problem and patients in intensive care are more vulnerable, requiring strict control measures and early identification. Currently, clinical culture materials are used to identify the bacterial agent, but saliva culture is not validated, which has great clinical relevance because it participates in several pathophysiological processes. The aim of this study was to validate saliva culture in an intensive care unit environment, determining its diagnostic value for infection. For this purpose, the results of the 39-month surveillance cultures, from the database of a private hospital were evaluated. A total of 323 cultures were paired between saliva, tracheal secretions, blood and urine from patients who were hospitalized for more than 5 days. The search for correlations between the results was performed using the Spearman correlation test. Severity and evolution data were also correlated. It was possible to correlate the presence of *Klebsiella* spp. between blood culture and saliva culture in 25% of the results (r = 0.01) and the correlation between saliva and tracheal secretion was 33% (r = 0.33447) with p < 0.0001. In conclusion, saliva can be an excellent discriminator of systemic infections, and can be considered a useful culture in clinical practice.

## Introduction

Antimicrobial resistance represents an escalating global health challenge, giving rise to increased morbidity and mortality rates while extending hospitalization durations, thereby straining healthcare systems and amplifying expenses. According to the European Centre for Disease Prevention and Control (ECDC), multidrug-resistant microorganisms contribute to approximately 25,000 annual fatalities^1^. Furthermore, in North America, the Centers for Disease Control and Prevention (CDC) estimates that around 2 million individuals are afflicted by infections originating from multidrug-resistant bacteria^2^.

In the realm of nosocomial infections, a recurring menace is posed by the “ESKAPE” pathogens, which encompass *Enterococcus faecium, Staphylococcus aureus, Klebsiella pneumoniae, Acinetobacter baumannii, Pseudomonas aeruginosa,* and *Enterobacter* spp. These pathogens are so named because they seemingly “escape” the impacts of antibacterial medications, significantly curtailing available therapeutic choices^3,4^.

Patients in intensive care units are exceptionally susceptible to the dynamics of antimicrobial resistance due to their frequent state of immunosuppression, heightened colonization pressure, and the selective pressure exerted by the administration of broad-spectrum antibiotics. The rising prevalence of multidrug-resistant bacteria inevitably precipitates a shortage of effective treatment alternatives, culminating in antibiograms devoid of viable therapeutic solutions. Within the sphere of intensive and hospital care, *Klebsiella* spp. assumes critical significance owing to its clinical relevance, virulence, and pervasive presence, mandating a tailored clinical approach^5–7^.

The COVID-19 pandemic has had a discernible impact on antimicrobial resistance, notably affecting the prevalence of bacterial infections among both COVID-19-infected and uninfected patients. Notably, *Klebsiella pneumoniae* has emerged as a prominent pathogen in COVID-19 patients admitted to the intensive care unit (ICU), with a significant contribution to bloodstream infections^8^. Consequently, preventive measures have assumed paramount significance in controlling bacterial infections and curtailing the dissemination of multidrug-resistant bacteria^9–11^.

Within the realm of surveillance policies, the monitoring of bacteria and their resistance patterns traditionally relies on clinical culture materials, encompassing sterile samples (such as blood, bone, cerebrospinal fluid, peritoneal fluid, pleural fluid, and synovial fluid) and non-sterile samples (including urine, sputum, tracheal secretions, and wound samples). Surveillance cultures from sites like the rectum, perianal region, axillary region, and nasal passages may not necessarily signify an active infection but rather serve as potential transmission reservoirs and risk factors for infection emergence, influencing the choice of antibiotic therapy^12^.

One intriguing avenue for monitoring bacterial infections lies in the oral microbiota, which ranks as the second most diverse microbial community in the human body after the gastrointestinal tract^13^. The oral microbiota plays a role in the pathophysiology of various diseases, including aspiration pneumonia^14^, type II diabetes, pancreatic cancer, and pediatric Crohn's disease^15^. Additionally, suboptimal oral hygiene heightens the risk of bacteremia associated with infective endocarditis, underscoring the intricate connection between the oral microbiota and bloodstream^16^.

Saliva stands out as a safe and non-invasive source of potentially valuable information, facilitating health status assessment and disease detection^17^. Saliva was reported in the literature as a diagnostic fluid (Malamud 2011) of bacterial and fungal infections (Arinawati and Novianti 2022). Nonetheless, the use of salivary culture in clinical practice within an ICU environment remains unvalidated, whether for surveillance or therapy guidance. Given saliva's attributes as an excellent culture medium for diverse bacteria^18^, a hypothesis emerges: monitoring the oral microbiota may lead to the development of control, decolonization, and treatment guidance strategies for multidrug-resistant bacteria. Therefore, the objective of this study is to evaluate the diagnostic value of saliva culture within an intensive care unit setting, assessing its role as an etiological factor for infection and colonization by *Klebsiella* spp.

## Material and methods

The results of surveillance cultures from the period 01/16/2018 to the period 06/04/2021 paired (collected at the same time) including saliva, blood, tracheal secretion and urine materials were evaluated. The data were obtained of the database of the Health Care Related Infection Control Service (SCIRAS) of Policlin Hospital (São José dos Campos, state of São Paulo, Brazil). These data pertained to surveillance cultures in the ICU. Additional data were collected from medical records (PHILIPS. Tasy, Hospital management system, 2019, USA) and the Epimed System (Epimed adult ICU monitor system, BR) to explore statistical correlations.

This study was performed in accordance with the Declaration of Helsinki, after obtaining authorization of the ethics committee involving human beings of the Institute of Science and Technology of São Paulo State University (ICT-UNESP), (CAAE 75033317.7.0000.0077). All experiments were performed in accordance with relevant guidelines and regulations. Informed consent was obtained from all participants and/or their legal guardians in this study.

### Surveillance cultures used

Surveillance cultures aim to assess the tendency of bacterial growth and to monitor the hospital microbial resistance profile by providing periodic reports. At Hospital, samples of saliva, secretion, blood and urine are periodically collected from patients admitted to the ICU. In mechanical ventilation cases, tracheal secretions are collected to monitor the bacterial profile by the Control of Health Care Related Infections (SCIRAS) internal surveillance protocol. Collections are obtained from 100 patients each semester. In this study, the inclusion criteria for the collection were patients with more than 5 days of hospitalization in the ICU of Hospital. Exclusion criteria: samples from patients who were hospitalized in the ICU for less than 5 days, as well as samples that did not show growth of microorganisms were not included in these analyses.

### Collection of surveillance cultures

Cultures were collected in paired saliva, blood, urine and tracheal secretions. Saliva collection was performed in small samples using a 1 mL syringe near to the exit of the salivary duct, between the lower first and second molars, then, it was identified by the health professional, placed in an appropriate container (Universal Sterile Bottle) and sent to microbiology sector of the Clinical Analysis Laboratory, where they were seeded and analyzed.

Blood culture collections were obtained by puncture with removal of 10 mL of blood at 2 different puncture sites (venipuncture, central catheter collection), these were packaged in appropriate blood culture flasks (BD bactec plus Aerobic/F Culture Vials, Becton, Dickson and Company, USA) and analyzed in the same laboratory.

Urine collections were performed depending on the patient's profile. In collaborative patients not probed, they were collected after cleaning the genital region with aqueous chlorhexidine or washing with soap and water, discarding the first jet and collecting from 20 to 50 mL; in uncooperative patients who are not using an indwelling urinary catheter, a urinary relief catheter was performed (a smaller probe than after urine collection was removed), discarding the first jet and collecting 20–50 mL; in previously probed patients, it was collected through a specific puncture in an appropriate place for puncture in the indwelling catheter of 20–50 mL. The material was stored in a sterile universal collector flask sent for analysis in the same laboratory. Tracheal secretion was collected only in patients on invasive mechanical ventilation through aspiration of tracheal secretions through the orotracheal tube, collected approximately 5 mL, stored in a specific bottle (Transbac C) and sent for analysis in the same laboratory.

### Analysis of cultures

The materials were homogenized and seeded in culture media Agar MacConkey (Laborclin, Brazil), Agar Blood (Laborclin, Brazil) and Tryptic Soy Broth “TSB” (Laborclin, Brazil). The plates and the broth were kept for 18–24 h at 37° until bacterial growth. The bacterial samples were identified using the API®/ID32 method, with biochemical tests and subsequently the bacterial resistance profile was carried out using the BD Phoenix NMIC/ID panel (Becton, Dickson and Company, USA), and the antibiogram was analyzed by the automated method BD Phoenix M50 (Becton, Dickson and Company, USA).

### Data collection

Data were gathered from the database of the Health Care Related Infection Control Service (SCIRAS) of Hospital. Relating to patient identification data (date of admission, gender, age and pathology), prognostic indexes (APACHE II and SAPS III), hospital mortality and if, during culture collection, there were a use of such invasive devices including the presence of mechanical ventilation, presence of central venous catheter and indwelling urinary catheter, collected through the electronic medical record system (PHILIPS. Tasy, Hospital management system, 2019, USA) and Epimed System (Epimed system monitor adult ICU, BR).

An Excel table was formatted to compile identification data (record number, patient name, age, gender, date of hospitalization, date of admission to the ICU, date of examination collection and hospitalization diagnosis), prognostic indexes that were commonly used in intensive care (Apache II, Saps III), mortality outcome in that hospitalization and use of invasive devices (mechanical ventilation, central venous catheter and indwelling urinary catheter) which increase the chance of colonization.

Data from culture results were distributed according to the type of material in the following sequence: saliva culture, tracheal secretion, peripheral blood culture, central blood culture, combined blood culture (meeting of peripheral and central blood cultures) and urine culture. Columns were formed to fill in as binomial data (1 or 0), 1 representing that it agreed with the column heading and 0 representing that it was not in agreement with the column heading.

Finally, a search was made for correlations between the results of bacterial and fungal cultures performed on species and genera unified in a data table (*Acinetobacter baumannii, Achromobacter* spp.*, Citrobacter Braaki, Citrobacter Freundil, Citrobacter koseri, Corynebacterium urealyticum, Enterobacter aerogenes, Enterobacter cloacae, Enterococcus faecalis, Enterobacter* spp., *Escherichia coli*, *Klebsiella oxytoca, Klebsiella ozaenae, Klebsiella Pneumoniae, Klebsiella* spp.*, Moraxella* spp.*, Morganella morganii, Proteus mirabilis, Proteus vulgaris, Providencia stuartii, Pseudomonas aeruginosa, Serratia marcencens, Staphylococcus aureus, Staphylococcus cohnii, Staphylococcus capitis, Staphylococcus coagulase negative, Staphylococcus lugdnensis, Staphylococcus epidermidis, Staphylococcus gallinarum, Staphylococcus haemolyticus, Staphylococcus hominis, Staphylococcus epidermidis, Staphylococcus saprophyticus, Staphylococcus simulans, Stenotrophomonas maltophilia, Streptococcus agalactiae, Ochrobactum anthropi, Candida albicans, Candida firmetaria, Candida freundii, Candida glabrata, Candida krusei, Candida parapsilosis, Candida tropicalis, Candida* spp. *Candida viswanathii, Candida utilis, Geotrichum* spp.*, Trichosporon asahii*) and on biological material obtained from 4 sites (saliva, blood, tracheal secretion and urine).

### Statistical analysis

#### n-sample calculation

The sample size was calculated to demonstrate the correlation, the a priori calculation was done through the derivation of the formula of the maximum estimate error, which was defined as E > 0.06. For such a procedure, the sample design dispenses with the correction factor for finite population^19^. With a significance level set to a value not greater than 0.05 and assumed to approximate the binomial distribution of proportions to a normal distribution, the following rule was followed: if the sample number (*n*) multiplied by the proportion of failures (*q*) is greater than or equal to 5 (*n x q* > *5*) and at the same time that the sample number (*n*) multiplied by the success proportion (*p*) is also greater than or equal to 5 (*n x p* > *5*), the distribution can be assumed by the normal curve. *P* = *q* = *0.5* was treated, which allows us to obtain a minimally sufficient sample to the detriment of the absence of previous data for the estimate. The n-sample was estimated at 267 observations.

#### Search for correlations

The search for correlations between the results of bacterial and fungal cultures performed in species and in biological material obtained from 4 sites (saliva, blood, tracheal secretions and urine) will be explored using the Spearman correlation test. The bacterium to be studied was the species of *Klebsiella* spp., they are the most prevalent in intensive care universally, species related to high mortality and are often multidrug resistant bacteria, with the highest prevalence in the Policlin ICU.

Spearman's correlation test is a non-parametric test that determines the degree of association between two variables arranged in ordered ranks. Through the coefficient (Rhô), we can estimate the strength and direction of a linear correlation with admission of small linearity leaks. As a result, the predictability of correlation between cultures. The tests were processed using Software OriginPro 9.5 (MicroCalTM, Northampton, MA, USA) and Bioestat 5.3 (University of Illinois, USA).

## Results

### Profile of patients and microorganisms

The results of surveillance cultures from the period 01/16/2018 to the period of 06/04/2021 paired (collected at the same time) between saliva, blood, tracheal secretion and urine materials were evaluated. From this sample, a total of 292 saliva cultures, 314 combined blood cultures (which could be blood through venipuncture, collection through the central venous catheter and sometimes 2 samples, but considered only one sample when positive), 111 results of tracheal secretion culture and 284 urine culture results were obtained.

Assessing the profile of patients, demographic data showed 51% of patients were male, the most prevalent age group was 61–80 years old with 37% of patients, the occupancy rate was 80%, the mortality rate was 10%, the mean Apache II was 6.83, SAPS III was 13.84, and the Expected/Observed Mortality Rate − 1 was 1.20. While the profile of the ICU patients who stayed for a period longer than 5 days, 50.1% were male, the most prevalent age group was mean of 70.5 years, mean Apache II was 14.45 and SAPS 3 was 34.51 and mortality was 34%.

The most frequent microorganisms isolated in saliva cultures was *Klebsiella* spp. with 54% of the isolates, followed by *Pseudomonas aeruginosa* 19%, *Acinetobacter baumannii* 7%, *Escherichia coli* with 4% and *Candida albicans* with 3%, another 13%. As demonstrated by the high prevalence of *Klebsiella* spp., the results of paired cultures between saliva and blood cultures with growth of *Klebsiella* spp. were selected for correlations in a total of 285 test results. As a control group to delimit the strength of the correlation, the relationship between tracheal secretion and blood was chosen, with 111 paired results.

### Quantification of positive cultures for Klebsiella spp.

In the sample of 285 paired cultures between saliva and blood, growth of *Klebsiella oxytoca* was observed in 10 saliva cultures and 1 blood culture, while *Klebsiella pneumoniae* was identified 107 times in saliva cultures and 23 in blood cultures and *Klebsiella* spp. 117 times in saliva and 24 in blood as shown in Table 1. Chosen to evaluate tracheal secretion and blood culture samples to serve as a basis for comparison between association results as tracheal secretion is a widely used culture. In the database, 111 paired cultures of tracheal secretion and blood cultures were found, 40 times *Klebsiella pneumoniae* were found in the tracheal secretion, and 71 negative results were found, while in the blood cultures, 14 times *Klebsiella pneumoniae* and 97 negative results were found in the paired sample, as shown in Table 2. A total of 102 paired cultures were found between saliva and tracheal secretions. Growth of *Klebsiella pneumoniae* was observed in 46 cultures of saliva and 35 cultures of tracheal secretion and *Klebsiella* spp. 47 times in saliva and 35 in tracheal secretion, as shown in Table 3.

**Table 1:** *Klebsiella* spp. frequency in 285 paired cultures -blood and saliva- from patients with more than 5 days in the ICU.

Bacteria	Saliva	Blood
*Klebsiella oxytoca*	10	1
*Klebsiella pneumoniae*	107	23
*Klebsiella* spp.	117	24
Negative	168	167

**Table 2:** *Klebsiella* spp. frequency in 111 paired cultures -blood and tracheal secretion- from patients with more than 5 days in the ICU.

Bacteria	Tracheal	Blood
*Klebsiella oxytoca*	0	0
*Klebsiella pneumoniae*	40	14
*Klebsiella* spp.	40	14
Negative	71	97

**Table 3:** *Klebsiella* spp. frequency in 102 paired cultures -Saliva and tracheal secretion- from patients with more than 5 days in the ICU.

Bacteria	Saliva	Tracheal
*Klebsiella oxytoca*	1	0
*Klebsiella pneumoniae*	46	35
*Klebsiella spp.*	47	35
Negative	55	67

### Correlation search

- After identifying the number of positive cultures, the search for correlations between two cultures was performed using Spearman's statistical test to determine the degree of association between cultures through the coefficient (Rhô), estimating the strength and direction of a linear correlation.
- In the evaluation between saliva and blood cultures, a coefficient of 0.2531 was found in *Klebsiella* spp. (p < 0.001), *Klebsiella oxytoca* had a coefficient of 0.34969 (p < 0.001), and in *Klebsiella pneumoniae*, the coefficient was 0.2655 (p < 0.001), indicating a linear correlation in both species, as shown in Table 4.
- When comparing saliva and tracheal secretion, Klebsiella spp. had a coefficient of 0.33447 (p < 0.001), and in *Klebsiella pneumoniae*, the coefficient was 0.30331 (p < 0.001), as shown in Table 5.
- Correlation between saliva and mortality outcome was performed, revealing a coefficient of 0.12247 (p value = 0.02774), which was higher than the correlation observed in blood samples, with a coefficient of 0.0892, although it lacked statistical significance.
- In contrast, there was an association between tracheal secretion and a higher mortality coefficient of 0.2169 (p value = 0.001), as shown in Table 6.

**Table 4:** Spearman correlation test result between *Klebsiella oxytoca*, *Klebsiella pneumoniae* and *Klebsiella* spp. in blood and saliva cultures.

Bacteria	Coefficient r (Rhô)	p value
*Klebsiella oxytoca*	0.34969	< 0,0001*
*Klebsiella pneumoniae*	0.26552	< 0,0001*
*Klebsiella* spp.	0.25313	< 0,0001*

*Statistically significant. Number of correlations tested: 12,270.

**Table 5:** Spearman correlation test between *Klebsiella pneumoniae* and *Klebsiella* spp. in saliva and tracheal secretion cultures.

Bacteria	Coefficient r (Rhô)	p value
*Klebsiella pneumoniae*	0.30331	0.00001*
*Klebsiella* spp.	0.33447	0.00001*

*Statistically significant. Number of correlations tested: 12,270.

**Table 6:** Spearman correlation test result between *Klebsiella* spp. in saliva, blood and tracheal secretion cultures and deaths.

Material	Bacteria	Death (Rhô)	p value
Saliva	*Klebsiella pneumoniae*	0.13194	0.01767*
	*Klebsiella* spp.	0.12247	0.02774*
Tracheal secretion	*Klebsiella pneumoniae*	0.2169	0.00008*
	*Klebsiella* spp.	0.2169	0.00008*
Blood	*Klebsiella pneumoniae*	0.0998	0.07328
	*Klebsiella* spp.	0.0892	0.10958

*Statistically significant. Number of correlations tested: 12,270.

## Discussion

In the realm of intensive care, infections stand as the leading causes of mortality, encompassing sepsis and healthcare-associated infections. Cultures play a pivotal role in this context, aiding in the identification of responsible microorganisms, guiding treatment strategies with specific antibiotics, and implementing environmental control measures like isolation to prevent inter-patient transmission^20^. Clinical cultures entail the collection of material samples to facilitate the observation of bacterial growth. In medical practice, they serve as essential diagnostic tools for identifying infections, assessing patient and hospital microbiota to gauge bacterial profiles and their resistance patterns against existing antibiotics. This ongoing monitoring assumes critical importance, especially considering the prevailing global pandemic of multidrug-resistant pathogens. Notably, the development of new antibiotics has not kept pace with the rapid evolution of microbial resistance^12,21,22^.

Within the intra-hospital setting, a variety of clinical culture materials are employed. They can be categorized into sterile samples (such as blood, bone, cerebrospinal fluid, peritoneal fluid, pleural fluid, and synovial fluid) which ideally should not exhibit bacterial growth; any growth would indicate infection. On the other hand, non-sterile samples (including urine, sputum, tracheal secretions, and wound specimens) are utilized in conjunction with other data to determine whether they signify an infection or mere colonization – denoting the presence of bacteria without active infection development. Additionally, surveillance cultures (derived from the rectal, perianal, axillary, and nasal regions) may not be directly linked to the disease's pathophysiological processes, but they do indicate patient colonization. These cultures are instrumental in monitoring bacterial growth trends within the hospital environment, providing insights into bacterial profiles, and enabling the establishment of strategies for isolation and control to curb the spread of multidrug-resistant microorganisms. While these isolates may not always denote a confirmed infection, they serve as potential reservoirs for transmission, pose a risk factor for infection emergence, and play a role in determining antibiotic therapy choices^3,12^.

Saliva culture, despite being commonly utilized in outpatient clinical settings such as dental offices, has yet to find its place in intensive care and hospital practices, either as a diagnostic tool or in the form of surveillance cultures. However, it's worth noting that the aspiration of oral secretions into the upper airways is often the initiating event in many cases of pneumonia in intensive care, underscoring the significance of oral health in this context. Additionally, poor oral hygiene has been linked to endocarditis. Considering that an average of 1 billion bacteria can be found in each drop of saliva, and during hospitalization, the oral bacterial flora can undergo modification and colonization by Gram-negative enterobacteria like Klebsiella spp., exploring the potential of saliva culture becomes pertinent^13,14,23^.

This study proposes an innovative approach by examining the correlation between saliva culture (a non-sterile site not typically used in hospital settings) and blood culture (a sterile and widely accepted site of clinical significance, denoting infection), specifically concerning the presence of *Klebsiella* spp. in critically ill patients. This research aims to validate the utility of saliva culture in hospital clinical practice, particularly within intensive care units, where patients are particularly vulnerable to bacterial translocation, infections, and heightened mortality. This pragmatic study relies on real-world database analysis and focuses on a specific subset of bacteria. The selection of *Klebsiella* spp., including *Klebsiella pneumoniae* and *Klebsiella oxytoca*, stems from their frequent occurrence in intensive care clinical scenarios and their noteworthiness as microorganisms causing diseases and mortality in this context. Extending this relationship to other bacterial classes would necessitate a larger patient cohort and a longer study duration, given the lower frequencies of other bacterial types in this setting.

The study's inclusion criteria involved the utilization of surveillance cultures performed biannually at Policlin Hospital. These cultures were conducted on patients with more than 5 days of hospitalization in the intensive care unit, with the primary objective of monitoring changes in the hospital's bacterial flora, which is known to evolve during a patient's hospital stay^24^. Notably, hospital microorganisms start to become detectable from the fourth day onwards^25^. The comprehensive nature of these criteria proved valuable in distinguishing the study population, which encompassed more severe patients with an established hospital bacterial flora.

This selectivity in patient recruitment was instrumental in achieving the study's objective. General ICU mortality during the period under examination stood at 10%. However, when specifically considering the mortality of patients for whom cultures were collected, the rate surged to 34%. Furthermore, severity indices assessed at the time of hospitalization, such as the Apache II index (which increased from 6.83 to 14.45) and the SAPS III (which escalated from 13.84 to 34.51), demonstrated a marked elevation among this subset of patients. The study allowed for the inclusion of repeat patients who remained hospitalized for extended durations. This consideration was essential because the length of a patient's stay exposes them to selective pressures stemming from antibiotic use, the introduction of new medical devices like central venous catheters, peripheral devices, orotracheal tubes, and indwelling urinary catheters. Additionally, it considers the frailty that patients develop during their illness and the course of their hospitalization. As the saying goes, “the patient from last week is not the same as this week.” Patients who continue to be hospitalized for more than 5 days in the ICU and whose underlying pathologies have not shown improvement transition into a category of more vulnerable chronic critically ill patients.

The study’s correlation analysis focused on the bacteria species *Klebsiella oxytoca*, *Klebsiella pneumoniae*, and the genus *Klebsiella* spp., examining the relationship between salivary culture and blood cultures. The results revealed a significant correlation, with predictive capabilities varying for different *Klebsiella* species: for *Klebsiella oxytoca*, the prediction accuracy was 35%, for *Klebsiella pneumoniae*, it stood at 26%, and for the genus *Klebsiella* spp., it reached 25%. These correlations were found to be statistically significant at a level lower than 0.001, highlighting the importance of saliva as a culture material compared to more established sites like tracheal secretions. Notably, this strong correlation was not observed in tracheal secretion cultures, underscoring the superior significance of saliva as a culture material. Furthermore, the study explored the link between saliva culture and patient mortality, revealing a relationship with a 12% correlation, supported by a p-value of 0.002. Although considered a secondary analysis, these findings reaffirm the underappreciated importance of saliva culture in the clinical practice of intensive care.

It's worth noting that blood culture, while widely used, is not considered a gold standard due to variations in its positivity rates depending on the type and site of infection, ranging from 5 to 50%^26^. Additionally, its positivity can be impacted by concurrent antibiotic use, further highlighting the significance of the correlations found in this study.

The study's findings, particularly the lack of correlation between tracheal secretion and blood culture, underscore the complexity of infection mechanisms and suggest that tracheal aspiration alone may not account for bloodstream infections. Moreover, inflammation in the oral cavity has been linked to the dissemination of pathogenic microbes through the bloodstream to distant sites in the body, leading to systemic infections^27^. These observations emphasize the critical importance of oral health care for critically ill patients, not only in preventing bloodstream infections but also in controlling nosocomial infections, potentially averting mortality.

While nasal and anal swabs remain valuable tools in diagnosing and monitoring infectious diseases, including COVID-19, saliva-based screening offers a less invasive, more comfortable, and potentially more scalable alternative for routine surveillance, particularly in settings like ICU. As technology and research in this field continue to advance, it is likely that we will see the increased use of saliva as a valuable tool for early detection and control of infectious diseases in educational institutions and beyond (Medeiros da Silva et al. 2020). Although the difference in mortality related to the presence of *Klebsiella* spp. in saliva was relatively low (0.12), its significance remains noteworthy. This study raises the hypothesis that interventions targeting treatment and oral hygiene could potentially reduce mortality rates.

## Conclusion

This study highlights the significance of saliva culture, often overlooked in clinical practice, as an excellent discriminator for bloodstream infections caused by Klebsiella spp. It demonstrated the ability to predict approximately 25% of blood culture results, emphasizing its potential utility in clinical practice. The findings encourage a reconsideration of the role of saliva culture in healthcare settings and its potential impact on patient outcomes.

## Data Availability

The data used to support the findings of this study are available upon request with the corresponding author d.d.s.amjad@gmail.com.
